# Translation of remote control regenerative technologies for bone repair

**DOI:** 10.1038/s41536-018-0048-1

**Published:** 2018-04-17

**Authors:** Hareklea Markides, Jane S. McLaren, Neil D. Telling, Noura Alom, E’atelaf A. Al-Mutheffer, Richard O. C. Oreffo, Andrew Zannettino, Brigitte E. Scammell, Lisa J. White, Alicia J. El Haj

**Affiliations:** 1 Institute for Science and Technology in Medicine, Keele University, Stoke-on-Trent, ST4 7QB UK; 2 Centre for Biomolecular Sciences, University of Nottingham, Nottingham, NG7 2RD UK; 3 Bone and Joint Research Group, Centre for Human Development, Stem Cells and Regeneration, Faculty of Medicine, University of Southampton, Southampton, SO16 6YD UK; 4 Adelaide Medical School, Faculty of Health and Medical Sciences, University of Adelaide, and South Australian Health and Medical Research Institute, Adelaide, SA 5000 Australia; 5 Academic Orthopaedics, Trauma and Sports Medicine, University of Nottingham, Queen’s Medical Centre, Nottingham, NG7 2UH UK; 6 School of Pharmacy, University of Nottingham, Nottingham, NG7 2RD UK

## Abstract

The role of biomechanical stimuli, or mechanotransduction, in normal bone homeostasis and repair is understood to facilitate effective osteogenesis of mesenchymal stem cells (MSCs) in vitro. Mechanotransduction has been integrated into a multitude of in vitro bone tissue engineering strategies and provides an effective means of controlling cell behaviour towards therapeutic outcomes. However, the delivery of mechanical stimuli to exogenous MSC populations, post implantation, poses a significant translational hurdle. Here, we describe an innovative bio-magnetic strategy, MICA, where magnetic nanoparticles (MNPs) are used to remotely deliver mechanical stimuli to the mechano-receptor, TREK-1, resulting in activation and downstream signalling via an external magnetic array. In these studies, we have translated MICA to a pre-clinical ovine model of bone injury to evaluate functional bone repair. We describe the development of a magnetic array capable of in vivo MNP manipulation and subsequent osteogenesis at equivalent field strengths in vitro. We further demonstrate that the viability of MICA-activated MSCs in vivo is unaffected 48 h post implantation. We present evidence to support early accelerated repair and preliminary enhanced bone growth in MICA-activated defects within individuals compared to internal controls. The variability in donor responses to MICA-activation was evaluated in vitro revealing that donors with poor osteogenic potential were most improved by MICA-activation. Our results demonstrate a clear relationship between responders to MICA in vitro and in vivo. These unique experiments offer exciting clinical applications for cell-based therapies as a practical in vivo source of dynamic loading, in real-time, in the absence of pharmacological agents.

## Bone repair: Magnetic nanoparticle therapy helps fix bone injuries in sheep

A biomagnetic therapy that stimulates adult stem cells helps promote repair in a sheep model of bone injury. Alicia El Haj from Keele University, UK, and colleagues previously developed a technique for activating specific ion channel receptors on stem cells through the use of targeted magnetic nanoparticles and a small magnetic field, but they had not tried the method on anything larger than a mouse. Here, the scientists tested the technique on sheep with injuries to their leg bones. They designed a magnetic array compatible with a sheep leg which could stimulate the cells for repair. They then tagged bone marrow stem cells with the nanoparticles, implanted the tagged cells at the site of injury, and applied an external magnetic array around the leg. The therapy accelerated repair and enhanced bone growth  compared to non-magnetically enhanced stem-cell treatments.

## Introduction

Large skeletal defects resulting from trauma, tumour resection and disease, remain a largely unresolved clinical problem, requiring a bone tissue engineering solution.^1–3^ Typically, with standard clinical intervention, the repair of a bone injury is achieved within 6 weeks owing to the highly efficient repair mechanisms involved in fracture healing. However, in 10% of all cases in which the volume of bone loss is significant, an inadequate bone healing response leads to the formation of a non-union or segmental defect.^4–6^ This condition represents a significant clinical challenge affecting people of all ages with substantial socio-economic implications in terms of treatment and hospital costs.^7,8^ While autologous bone grafts are considered the gold standard to address the issue of non-union fractions, there remain associated limitations leading to the development of alternative stem cell-based or regenerative medicine therapies.^1,5,9,10^

Bone homeostasis, remodelling and fracture repair mechanisms are regulated by a process known as mechanotransduction, the conversion of physical forces acting on a cell to internal biochemical signals.^6,11–14^ Despite the many published in vitro studies identifying the need for mechanical conditioning of osteoblasts and their mesenchymal stem cell (MSC) precursors to drive osteogenesis and tissue maturation, few technologies have been successfully translated into pre-clinical studies of bone repair. While whole body rehabilitation programmes are routinely prescribed in a clinical setting, a technology of clinical human relevance which can translate physical stimuli into biological responses in a controlled and localised fashion has, to date, not been achieved. As such, mechanical stimuli are often lacking in stem cell-based therapeutic approaches for bone regeneration.^9,13^ This can impede stem cell differentiation in vivo and ultimately tissue synthesis, with a significant impact on the quality and quantity of bone formed thus affecting the clinical outcome of the treatment.^13^

We have developed a pioneering bio-magnetic technology (MICA; Magnetic Ion Channel Activation) designed to remotely deliver directed mechanical stimuli to individual cells in culture or within the body, to promote osteogenesis.^15–17^ By targeting specific mechano-sensitive ion channels on the cell membrane of MSCs with functionalised, biocompatible, magnetic nanoparticles (MNPs), the opening of the ion channel can be controlled with an oscillating external magnetic field. The movement of the particle creates a pico-newton force that is transferred to the ion channel to which the MNPs have attached, propagating the mechanical stimulus via mechanotransduction pathways inside the cell.^15–18^ One such mechano-sensitive ion channel is TREK-1, a potassium channel whose function is to maintain membrane potential and plays a critical role in the mechanotransduction signalling pathways in bone.^17^

In our earlier in vitro studies, we demonstrated using an electrophysiological patch clamping model that we could open and activate the 6 His tagged TREK-1 channel expressed in the membrane of cells using remote mechanical movement of Ni^2+^ labelled MNPs.^17^ Importantly, these studies demonstrated the specificity of this technique as no TREK-1 channel activation was observed when MNPs were coated with RGD (Arg–Gly–Asp) peptide, or when magnetic fields were applied in the absence of MNPs. Furthermore, we went on to demonstrate that we could deliver forces in the region of 8–15 pN onto the membrane channels using remotely controlled MNPs which lead to the differentiation of bone marrow-derived stromal stem cells in vitro.^15^ We have generated further proof of concept data showing activation of the TREK-1 ion channel in 2D models of osteogenesis,^15^ 3D cell-seeded constructs in vitro, and ex vivo bone tissue engineering models.^13^ Our preliminary study in a small animal model, showed controlled differentiation of bone marrow stromal stem cells in hydrogel capsules implanted subcutaneously in the dorsal region of nude mice.^19^

This manuscript describes the translation of this technology to a relevant pre-clinical ovine bone defect model to explore the therapeutic potential of MICA for bone repair. Our aim is to demonstrate the relevance of MICA technology for use as a clinical therapy, and a potential solution for the control of therapeutic donor cells in regenerative medicine applications. In addition, we consider the individual variation in responses between sheep donors to further understand “good” and “poor” responders within an ovine population.

## Results

### STRO-4 positive oMSCs from all donors demonstrate tri-lineage differentiation capacity

**Fig. 1:**
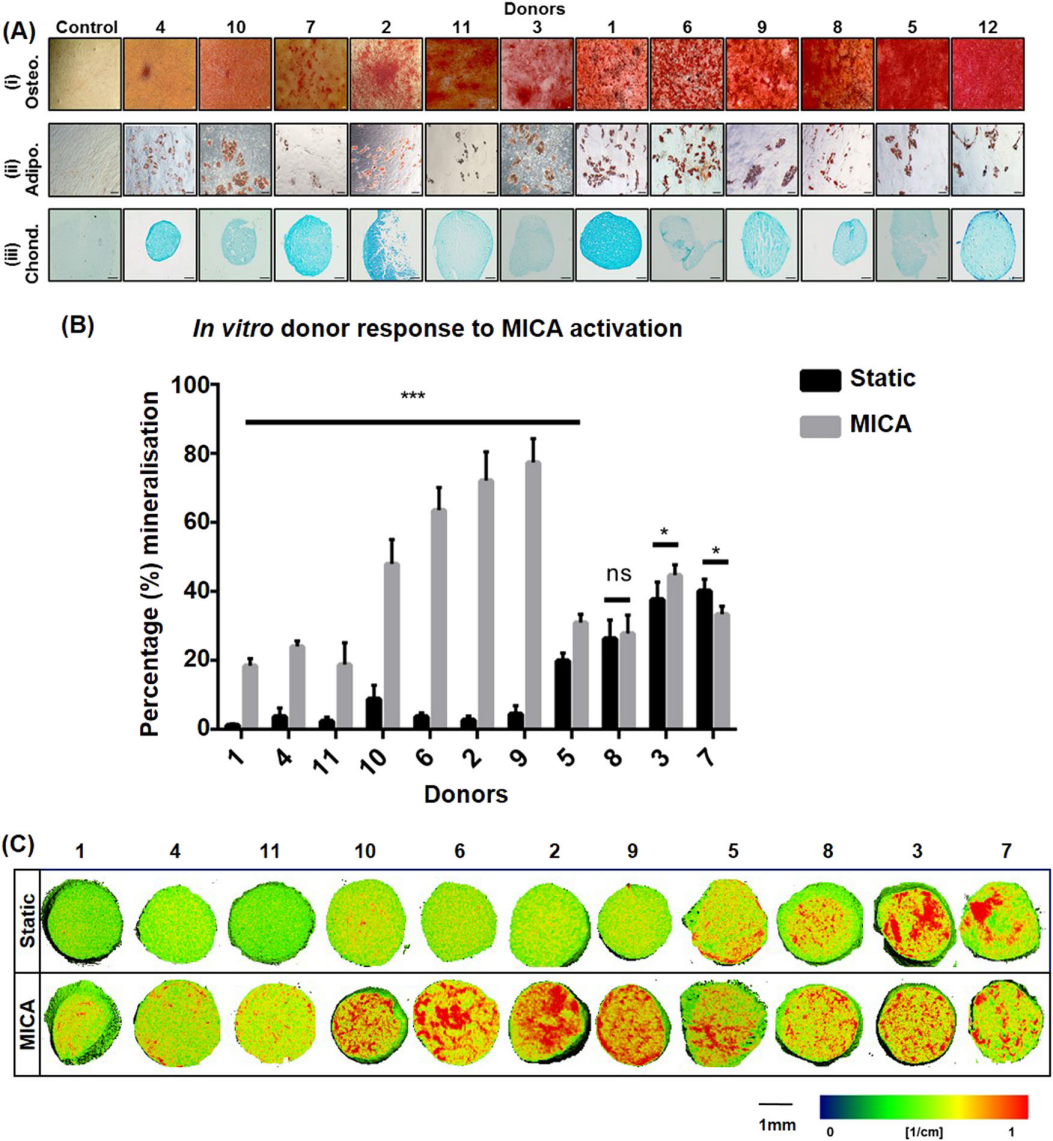
In vitro assessment of donor cell differentiation potential. **a** Comparative tri-lineage differentiation of STRO-4 positive ovine mesenchymal stem cells (oMSCs) from 12 sheep donors. Images are presented in order of increasing differentiation potential for **a**, i Osteogenesis at day 28 (Alizarin Red staining) with corresponding **a**, ii Adipogenesis at day 14 (Oil Red O staining), **a**, iii Chondrogenesis at day 21 (Alcian Blue staining) and compared to a representative proliferation media control (*n* = 3), scale bars; 100 µm. **b** Quantification of in vitro donor response to MICA activation in 3D collagen hydrogel cultures assessed by Micro-CT at day 28 and compared to static controls. Data represents the average percentage mineralisation for donors 1–11 ± S.D. (*n* = 9). **c** Corresponding 2D slices showing mineralisation (red regions) representing the central slice of the 3D hydrogel. Scale bar; 1 mm. Statistical significance is represented by *P < 0.05, ***P < 0.001 and ns is no significance

STRO-4 positive oMSCs (ovine mesenchymal stem cells) were characterised by their ability to undergo osteogenic, adipogenic and chondrogenic differentiation. Cells from all 12 donors (experiment 2, Table 1) were successfully differentiated towards all three lineages with marked donor dependent variation (Fig.1a).

**Table 1:** Experimental groups

Group	Cells	MNPs	Magnet	DiI stain	Number of defect	Time point
Experiment 1; Effect of MICA treatment on in vivo cell fate						
1 (MICA)	+	+	+	Yes	6	2 days
2	+	+	−	Yes	6	2 days
3	+	−	+	Yes	6	2 days
4	+	−	−	Yes	6	2 days
Experiment 2; Effect of MICA treatment on bone repair						
1 (MICA)	+	+	+	No	6	13 weeks
2	+	+	−	No	6	13 weeks
3	+	−	+	No	6	13 weeks
4	+	−	−	No	6	13 weeks
5 (ECM carrier alone)	−	−	−	No	6	13 weeks
6 (BG) Bone graft	−	−	−	No	6	13 weeks
7 (E) Empty	−	−	−	No	6	13 weeks

### Donors with lower osteogenic potential displayed an enhanced osteogenic response after MICA technology application in vitro

The response of each set of donor oMSCs (donors 1–11, experiment 2, Table 1) to MICA activation was assessed in a 3D collagen hydrogel culture system. Variable mineralisation levels were observed across donors exposed to an osteogenic environment and to MICA activation. Donors displaying low mineralisation levels in the static groups exhibited significantly enhanced osteogenesis following MICA activation (*P* < 0.001) (donors 1, 4, 11, 10, 6, 2, 9; Fig. 1b) with the fold-change increase ranging from 0.5-fold (donor 5) to 25-fold (donor 2). Donors with a stronger osteogenic response in static conditions were not influenced by MICA activation to the same extent (donors 3; *P* < 0.05, donor 8; ns). Finally, a key finding relevant to this study was that only donor 7 demonstrated a slight, but significantly negative response to MICA activation (*P* < 0.05). This data is supported by the density maps of each gel demonstrating regions of high density mineralisation as red (Fig. 1c).

### Design and development of the magnetic array for in vivo MICA activation

**Fig. 2:**
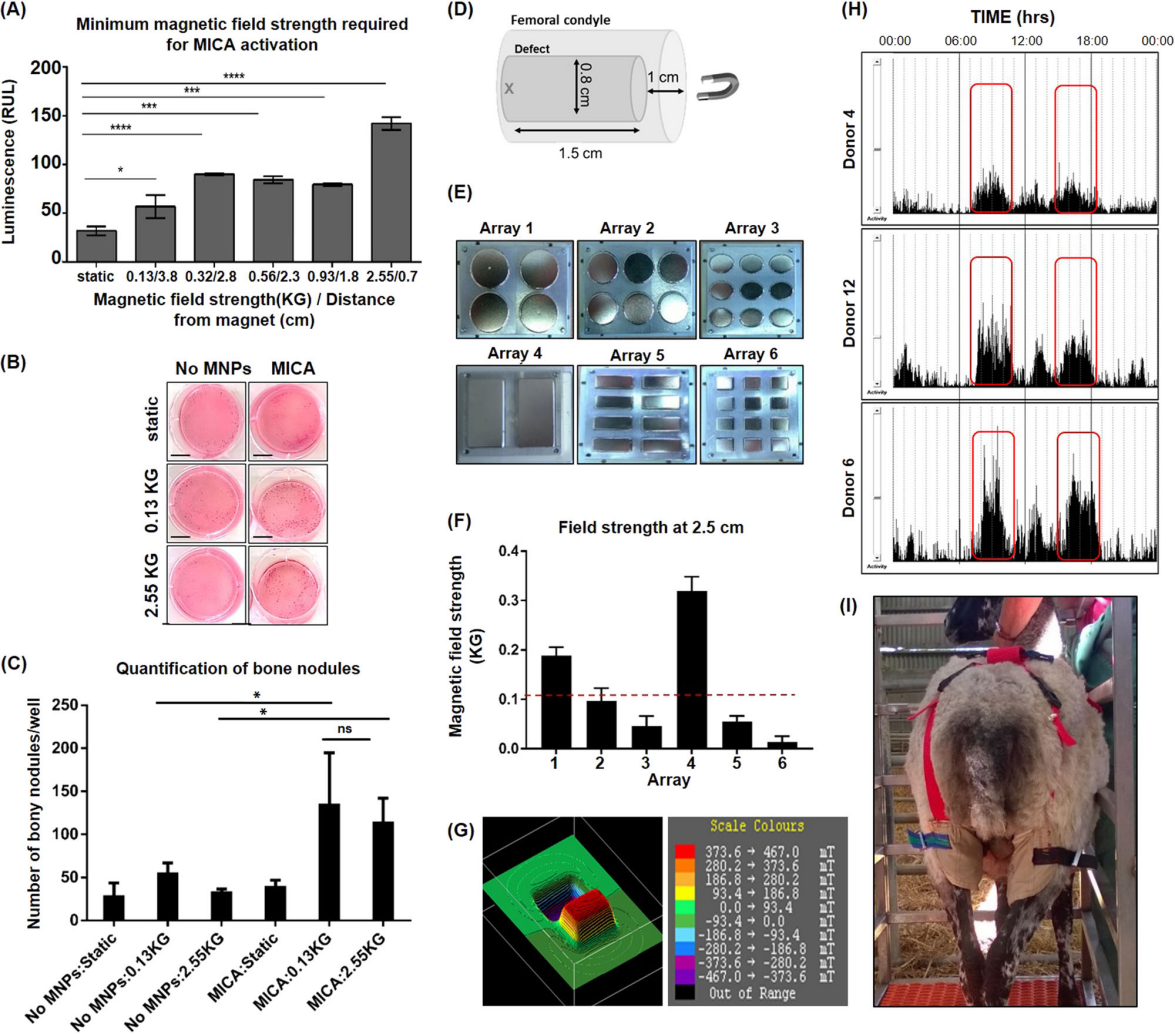
Design and development of a magnetic array for in vivo MICA activation. **a** Determining the minimum magnetic field strength required for cell activation. MICA activation of MNP-labelled HEK-293 NFΚ-β reporter cells at increasing magnetic field strengths (corresponding to a decrease in distance between cells and the magnetic array). Data represents the mean luminescence (RLU) ± SEM (*n* = 3). **b** MICA activation of MNP-labelled and unlabelled STRO-4 positive ovine mesenchymal stem cells (oMSCs) towards osteogenesis (Alizarin red staining) in 6-well monolayer cell culture plates at a field strength of 0.13 and 2.55 KG and compared to static and unlabelled controls (*n* = 3), scale bar; 1 cm. **c** Quantification of bony nodules generated in monolayer as a result of MICA activation at either field strength (0.13 and 2.55 KG) and compared to static and unlabelled controls. Data represents the average number of visible bone nodules across 3 wells of a 6-well plate. **d** Schematic representing the size and location of the defect within the femoral condyle relative to the position of the magnetic array. “X” marks the location of MNP-labelled cells furthest away from the magnet i.e 2.5 cm in the ovine model. **e** Fabrication of six magnetic arrays containing neodymium iron boron magnets of varying dimensions. **f** Comparative magnetic field strength from arrays 1–6 at a distance of 2.5 cm. Data represents the average magnetic field strength at six random points on each magnet per array ± S.D. Red dashed line represents minimum magnetic field strength (0.13 KG) required to activate cells. **g** 3D Magnetic profile of array 4 at a distance of 0.5 cm demonstrating alternating poles. **h** Accelerometer data for sheep donors 4, 6 and 12 highlighting most active periods (red boxes) within a 24 h period. **i** Picture of a sheep fitted with the adapted truss housing magnetic array 4 within the pouch corresponding to the location of the defect. Statistical significance is represented by *P < 0.05, ***P < 0.001 and ns is no significance

A vital component of this study was the development of a magnetic array compatible with the ovine model to enable activation of cells post-implantation. The external magnetic field strength required to activate MNP-labelled cells once implanted within the femoral condyle defect was determined in vitro using a HEK-293 NFK-β luciferase reporter cell line. Although greatest activation was achieved at the highest field strength, 2.55 KG (Kilogauss), cells stimulated with weaker fields (0.92, 0.56, 0.32, 0.13 KG) continued to demonstrate significantly enhanced activation compared to the static controls, albeit at reduced levels (Fig. 2a). The minimum magnetic field strength required for in vivo MICA activation was thus determined to be 0.13 KG. Downstream osteogenesis at field strengths 0.13 KG and 2.55 KG was validated in 2D monolayer cell cultures (Fig. 2b). This resulted in improved mineralisation in all MICA activated groups (Fig. 2c), as seen by the significant increase in the number of bony nodules produced compared to control groups (unlabelled and static condition) regardless of field strength (Fig. 2b, c). The schematic (Fig. 2d) represents the size and orientation of the femoral defect relative to the position of the magnetic array, defining the maximum working distance as 2.5 cm (“x” Fig. 2d).

Collectively, these data informed the primary design parameters of the array and were taken forward to fabricate six permanent magnetic arrays featuring magnets of varying dimensions and shapes (Fig. 2e). Arrays were validated against the primary design parameters, identifying arrays 1 and 4 as the only candidates capable of generating a field strength of 0.13 KG at 2.5 cm (Fig. 2f). Array 4 was selected for subsequent in vivo ovine studies. Two magnets were inserted into the aluminium frame with adjacent alternating poles to generate the field gradient required for MNP manipulation (Fig. 2g). Accelerometers were used to determine when sheep were most active as the changing magnetic field gradient was achieved with the movement of the sheep leg (Fig. 2h). Through monitoring the activity of 3 sheep over 7 days, 2 periods of increased activity were observed; 08:00–11:00 and 15:00–18:00 (Fig. 2h). Arrays were placed in a pouch fitted around the back legs of each sheep corresponding to the location of the defect (Fig. 2i) and worn in hours of peak activity.

### Surgical model

Surgery was tolerated well by all sheep without complications. No signs of adverse reactions to the ECM (extracellular matrix) hydrogel or MNP delivery were observed. C-reactive protein (CRP) levels were measured 2 days’ post implantation (experiment 1, Table 1; data not shown) revealing no deviation from baseline levels. After an initial adjustment period, animals appeared to tolerate the magnet truss well with no irritation of the fresh wound and, importantly, no impaired mobility.

### ECM construct remains intact and 50% of cells remain viable 2 days after implantation

**Fig. 3:**
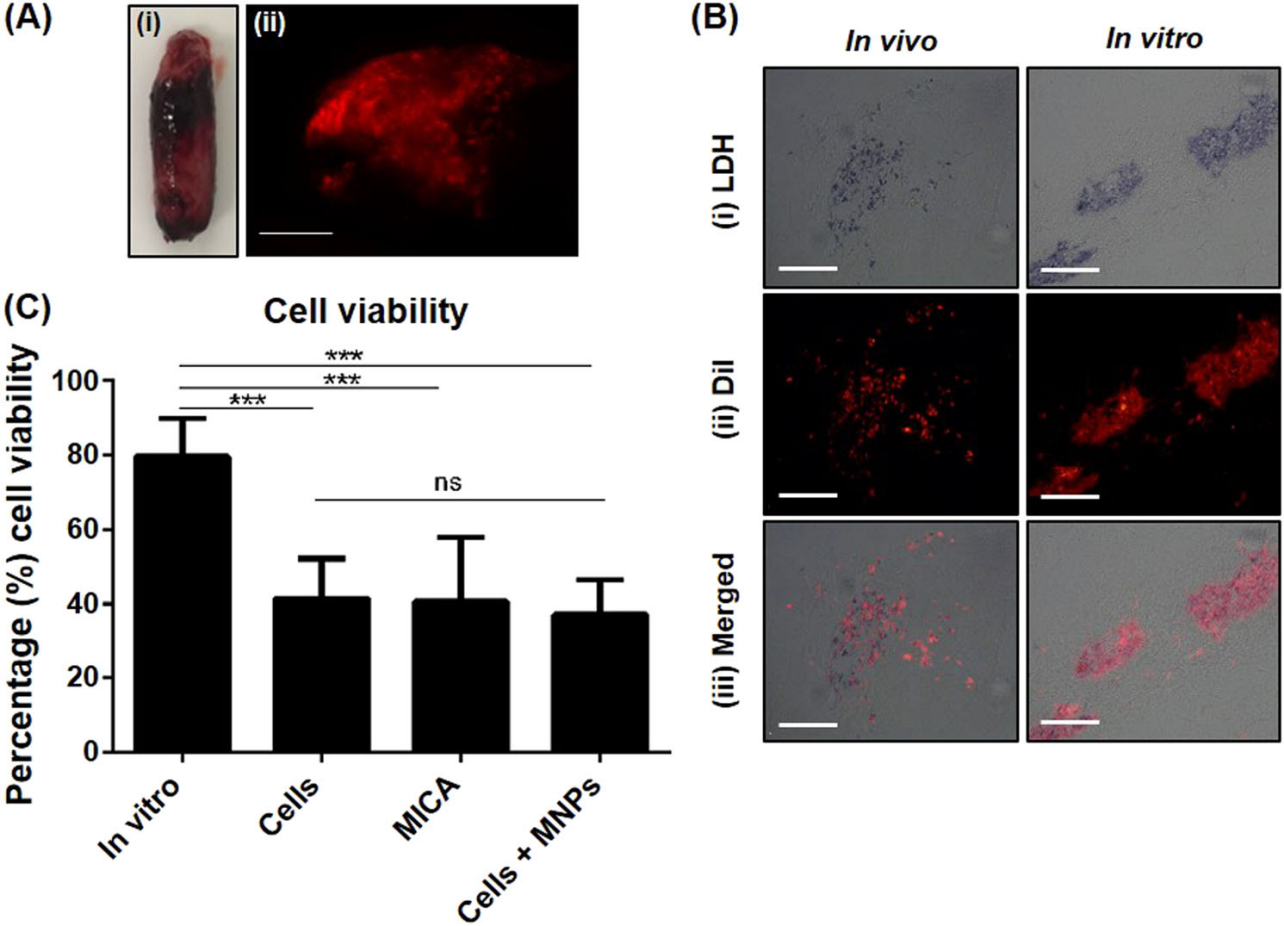
Assessment of oMSC fate 48 h post implantation. **a**, i Implanted ECM-constructs remained intact with **a**, ii delivered oMSCs (CM-DiI-stained; red fluorescence) visibly distributed throughout the implanted construct; scale bar; 2 mm. **b** Representative cryo-sectioned samples of the extracted in vivo construct and time-point matched in vitro controls constructs. **b**, i Viable oMSCs were identified by a distinct blue stain attributed to the LDH reaction. **b**, ii Implanted oMSCs were identified by red fluorescent staining. **b**, iii Viability of delivered cells was therefore determined by the co-localisation of blue and red-fluorescent stains. **c** Quantification of cellular viability for all in vivo groups (cells only, MICA and cells + MNPs) and compared to time-point matched in vitro controls. Data is presented as the average viability (proportion of duel LDH:DiI labelled cells relative to total DiI labelled cells) for 5 random sections where 10 independent FOVs were analysed per section for each sample ± S.D (*n* = 6). Statistical significance is represented by * where, ***P < 0.001 and ns is no significance

The short-term fate of delivered oMSCs and the impact of MICA activation on cell viability and construct integrity was assessed in experiment 1 (Table 1). Constructs were extracted fully intact (Fig. 3a i) 48 h post-implantation with CM-DiI labelled oMSCs (implanted oMSCs) clearly visible throughout (red fluorescence) (Fig. 3a, ii). An increase in construct stiffness was observed post-harvest when compared to in vitro controls, with the general size remaining wholly unchanged (6.4 ± 40.68 × 14.83 ± 1.2 mm) when compared to pre-implanted standard dimensions (8 × 15 mm). Lactate dehydrogenase (LDH) is an enzyme present in all living cells responsible for catalysing the reaction resulting in the blue staining of viable CM-DiI labelled cells (Fig. 3b). Quantification of LDH stained cells (Fig. 3c) revealed an approximate 50% loss in cell viability (*P* < 0.001) across all groups compared to the corresponding in vitro control, with no influence of MNP-labelling nor MICA activation.

### MICA treatment enhances early bone formation

**Fig. 4:**
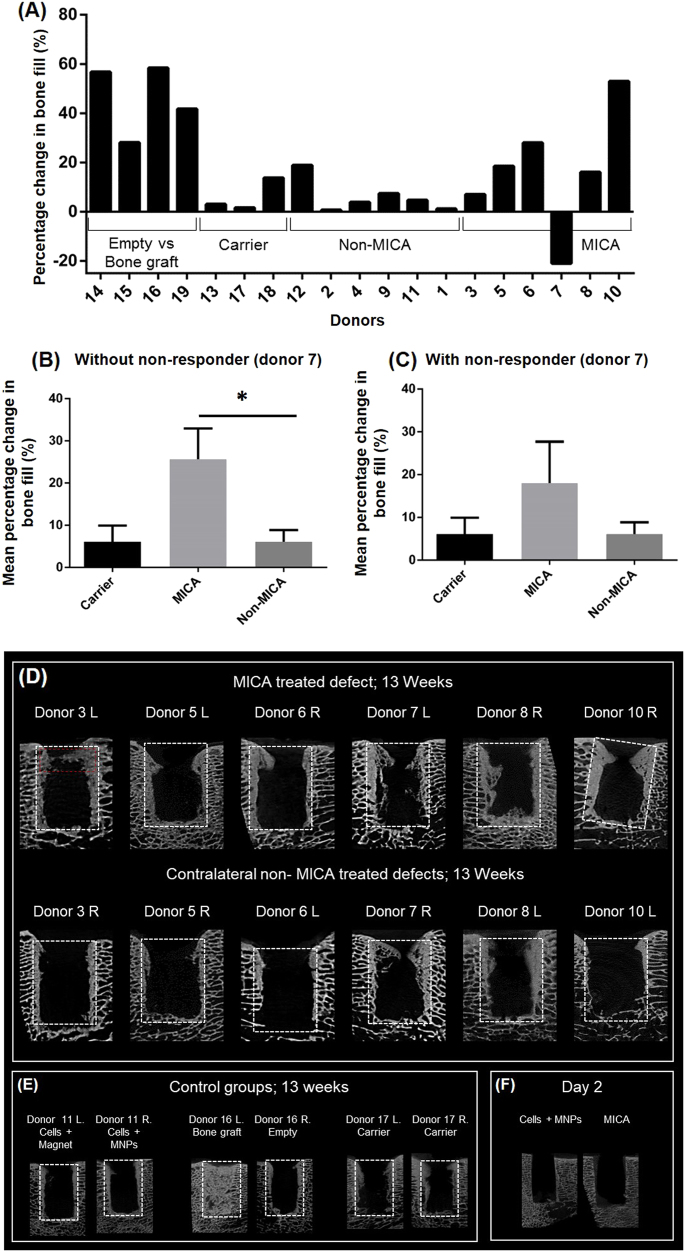
Micro-CT evaluation of bone repair at 13 weeks. **a** Percentage change in bone growth between defects of the same animal (*n* = 1). **b**, **c** Corresponding averaged percentage change for the same sheep (*n* = 6) either excluding or including donor 7, the non-responder respectively. **d** Representative Micro-CT slices for all 6 MICA treated sheep (donors 3, 5, 6, 7, 8 and 10) comparing the left (L) and right (R) defects of each sheep (MICA vs non-MICA) at 13 weeks. **e** Representative control groups include a non-MICA treated sheep (donor 11L & R), a positive control (donor 16L; bone graft), the negative control (donor 16R; empty defect), a carrier control (donor 17L & R) and **f** micro-CT images of a defect at day 2 treated either with MICA or non-MICA (cells + MNPs). White dotted box represents the analysed region of interest. Red dotted box represents region corresponding to histological analysis. Statistical significance is represented by *P < 0.05

Bone growth was evaluated by micro-CT at 13 weeks as an indication of early repair. To account for donor-dependent responses and eliminate biological variation, data was assessed on an individual sheep basis (Fig. 4a). This was achieved by comparing bone volume in the left and right defects of the same sheep and expressing this as a percentage change in bone volume. In this way, the effectiveness of two independent treatments can be assessed in the same animal which has been treated with an identical population of autologous cells. MICA treated defects repaired to a greater degree in comparison to the control defect of the same animal in five out of six sheep (Fig. 4a), with donor 7 identified as the non-responder. When grouped, an average improvement of 25 ± 6.5% is detected in MICA treated animals by excluding the single non-responder, donor 7 (*P* < 0.05) (Fig. 4b), and by 17.8 ± 8.9% by including donor 7 (Fig. 4c) compared to the non-MICA animals. In comparison, sheep treated either with a MICA-control group or the ECM carrier alone in both legs demonstrated little differences in the degree of repair between the two defects (6.5 ± 5.8% difference for non-MICA sheep and 6.1 ± 5.4% difference for ECM carrier control sheep).

**Fig. 5:**
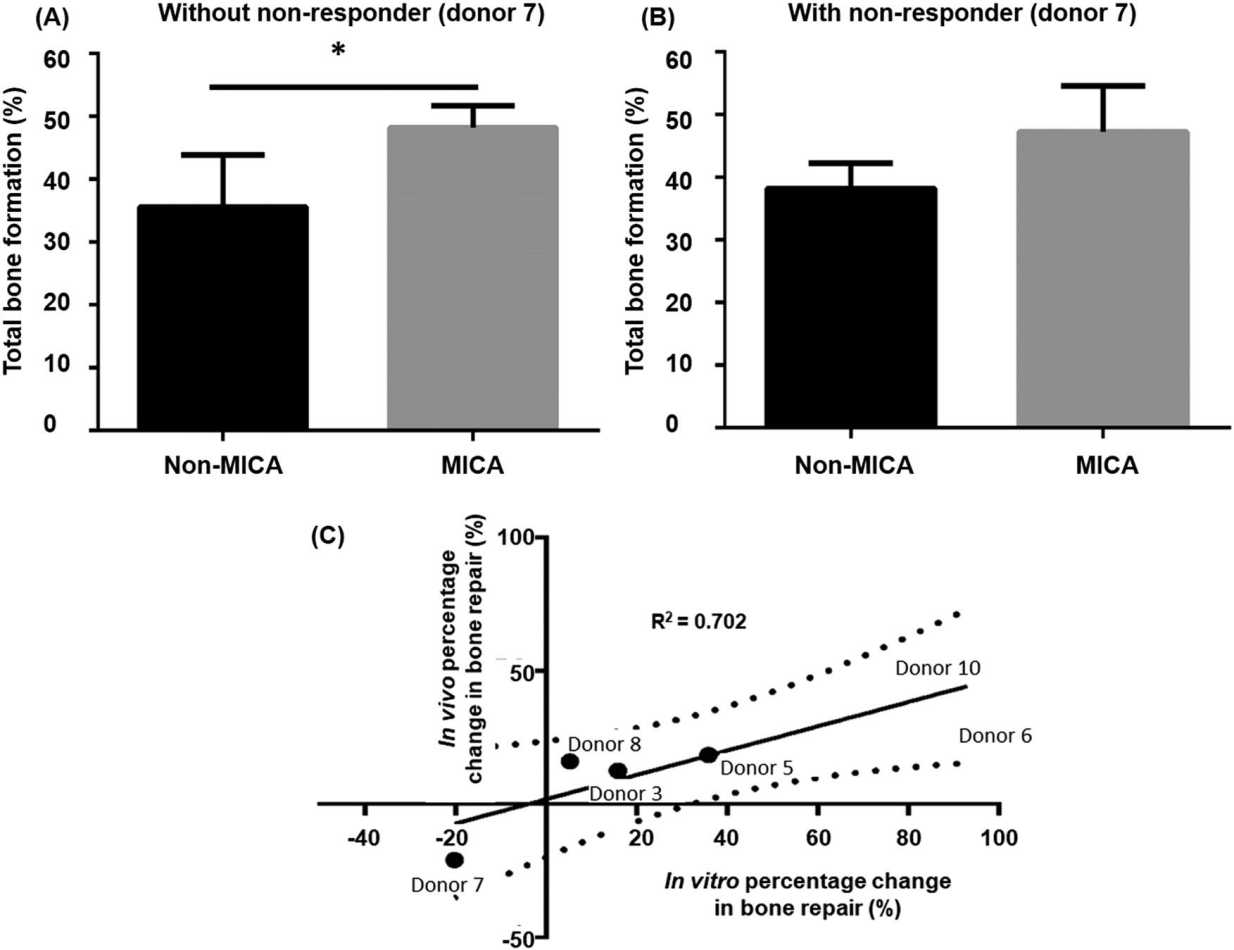
Continuation of Micro-CT analysis. **a**, **b** Averaged total bone formation comparing MICA treatment to the contralateral MICA control (non-MICA) for donors 3, 5, 6, 7, 8 and 10 either excluding or including donor 7 respectively. **c** Correlation of the in vitro and in vivo responses to MICA activation for donors 3, 5, 6, 7, 8 and 10 when comparing the percentage in change in mineralisation relative to donor matched static control and percentage change in bone fill relative to the non-MICA contralateral control leg of the same animal respectively. Dotted lines indicate the 95% confidence band. Line of best fit plotted with a *R*^2^ value of 0.7072

This data is supported visually by micro-CT images of defects from the same sheep, where greater bone growth is observed in the proximal (top) and peripheral (side) regions in MICA treated defects compared to the contralateral MICA-control defect of the same donor for donors 3, 5, 6, 8, and 10 but not for donor 7 (Fig. 4d). The gold standard treatment for large skeletal defects is typically autologous bone graft, which was used as the positive control in this study. In this short term study, this treatment group where autologous bone is implanted to fill the site can be seen to completely occupy the defect with autograft and autologous remodelled bone (Fig. 4e; donor 16L). Finally, bone growth is seen in all groups originating at the boundaries of the defect with new bone growth evident as regions of high density bone that is not seen in day 2 scans (Fig. 4f). Finally, considering the average population response, we demonstrate an increase in total new bone formation in MICA treated defects compared to non-MICA control groups (Fig. 5b), significant only when donor 7 is excluded (*P* < 0.05) (Fig. 5a).

### Good correlation between in vitro and in vivo donor response to MICA activation

Tracking the individual responses within the sheep enabled us to identify correlations between the good responders and the poor responders in vitro and in vivo. We observed a clear correlation (*R*^2^ = 0.7072; Fig.5c) between the in vitro performance (assessed as percentage change in mineralisation relative to the corresponding static control; Fig. 1b) and the in vivo bone fill (calculated as percentage change in bone fill relative to the non-MICA control leg of the same sheep; Fig. 4a) in this study.

### Bone of greater maturity is observed in MICA treated defects with enhanced recruitment of endogenous cells

**Fig. 6:**
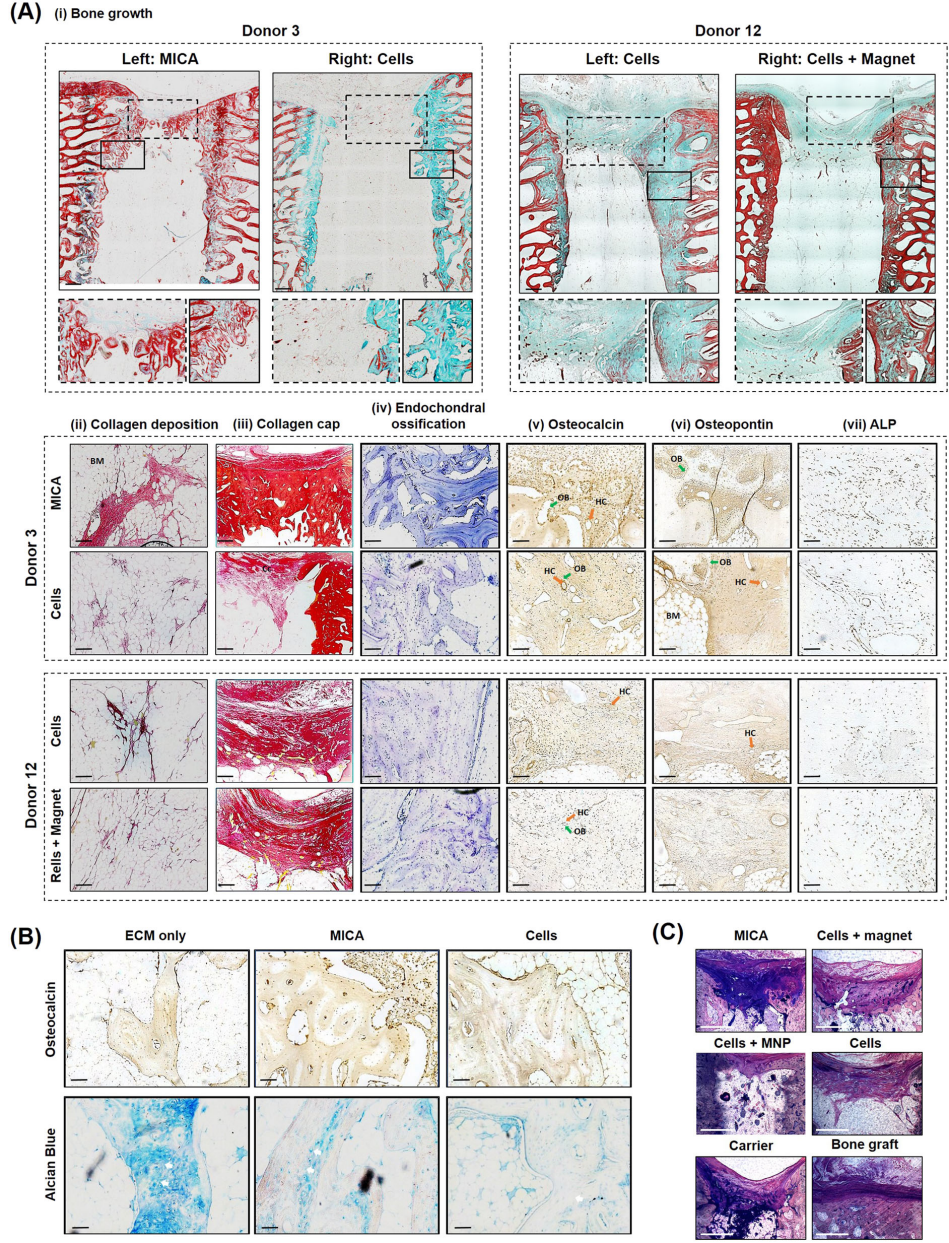
Histological evaluation of repair at 13 weeks. **a** Representative images from; Donor 3 (MICA animal) treated with MICA (left defect) and cells only (right defect) and Donor 12 (MICA-control animal) treated with cells only (left defect) and cells + magnet (right defect). Histological staining; **a**, i Masson-Goldner trichrome staining identifying new bone callus in green, osteoid steams in red and focused on bone outgrowth over the top of the defect and along the peripheral edges (inserts). **a**, ii and **a**, iii Picrosirius red staining of collagen rich structures in the central and proximal regions of each defect respectively. a, iv Toluidine blue staining identifying cartilage-like tissues rich in proteoglycans (indicative of bone growth via the endochondral ossification route) in purple. **a**, v Osteocalcin **a**, vi osteopontin and **a**, vii ALP (alkaline phosphatase) immuno-histochemical (IHC) staining at the proximal region of each defect. **b** Representative ECM-carrier, MICA and cell only sections stained for Alcian blue and Osteocalcin IHC demonstrating areas of cartilage like tissue (Alcian blue) and areas of mineralised tissue (osteocalcin). **c** Representative calcified sections from each group stained with paragon and toluidine blue staining; new bone growth is identified by light pink staining while fibrous tissue is stained deep purple. Scale bar represents 500 µm (**a**, i, **a**, iii),100 µm (**a**, ii, **a**, iv, **a**, v, **a**, vi, **a**, vii, **b**) or 1500 µm (**c**). Green arrow (OB); osteoblasts, orange arrow (HC); Haversian Canals, white arrows; hypertrophic chondrocytes, BM; Bone marrow. For further information on the anatomical location of each section, please refer to supplementary information, Fig. 3

Implanted constructs were not present at 13 weeks, with evidence of new bone structures visible in all groups. Differences in the amount, distribution and maturity of new bone was observed between donors and treatment groups (Fig. 6a). Although peripheral (side) bone growth from the surrounding trabeculae was observed in all groups (Fig. 6a, i), evidence of bone extensions across the defects was present only in the MICA group (Donor 3L; Fig. 6a, i) and lacking in the contralateral MICA-control defect (Donor 3R; Fig. 6a, i). Evidence of bone growth was also observed in non-MICA animals (Donor 12; Fig. 6a, i). Trabecular-like architecture was again evident only in the MICA defect with intense red osteoid staining surrounding new structural bone indicative of maturing bone and active osteogenesis (Donor 3L; Fig. 6a, i). Although complete union was not achieved over this time period, bone marrow-like tissue was present within defects of all groups, with collagen fibres dispersed throughout this matrix in all groups (Fig. 6a, ii). Toluidine blue staining highlighted the presence of new woven bone which is prominent in all groups, but higher intensity of staining was observed in MICA defects (Fig. a, iv). A collagen rich soft tissue structure is present in the proximal (top) region of each defect (Fig. 6a, iii) with immuno-histochemical analysis revealing key bone extracellular matrix components, osteocalcin (Fig. 6a, v) and osteopontin (Fig. 6a, vi), embedded within this collagen structure. Furthermore, this region was found to be rich in osteopontin-positive and osteocalcin-positive cells suggesting the presence of functional osteoblasts and osteocytes involved in bone remodelling. ALP immunohistochemistry again revealed functional osteoblasts distributed within this region at a greater cellular density in the MICA defects and was associated with active remodelling (Fig. 6a, vii). Overall, greater cellular density of osteopontin-positive cells were observed in the MICA-treated defect (Donor 3L) compared to the contralateral defect (Donor 3R) and to either defects of donor 12 in which a similar cellular density was observed (Fig. 6a, v). Early signs of remodelling were observed in this region with early structural Haversian Canals (orange arrow) appearing to develop, lined by osteoblasts (green arrow). Evidence of the remnant cartilaginous tissue and hypertrophic chondrocytes (white arrows) were observed in all groups including the ECM-carrier group (Fig. 6b). Furthermore, mineralisation of the cartilaginous tissue within cell based groups (MICA and cells only) appeared to have progressed further than the ECM treatment alone with regions of greater osteocalcin staining observed (Fig. 6b). Calcified histological sections demonstrate fibrous capping in the proximal regions in all groups (Fig. 6c). A large amount of callous was found at the edges of all defects, except in the bone graft group. Signs of osteons and osteocytes are present with borders of osteoblasts and a visible osteoid layer at the interface between new bone and fibrous tissue.

## Discussion

We describe an innovative remote bio-magnetic activation technique (MICA) which can be used to control the behaviour of MSCs in clinical stem cell-based therapies. Using an early stage pre-clinical ovine model, we show that targeted activation of the TREK-1 ion channel, present in oMSCs, can lead to initial enhanced repair in donor-matched controls. Evidence of early elevated new bone formation and increased bone outgrowth across the defect were observed in MICA-treated defects. Assessment of individual sheep, using internal controls to eliminate variations in the base-line levels of repair between sheep and donor stem cell behaviour, allowed for assessment of the early effects of MICA on defect repair and demonstrated a correlation in ‘good’ and ‘poor’ responders between in vitro and in vivo studies.

In recent years, MSCs have emerged as appealing therapeutic agents in the development of skeletal stem cell-based therapies and have demonstrated remarkable clinical potential. A limitation with using MSCs in clinical scenarios is the availability and expansion of these cells to therapeutic numbers. Typically, less than 0.001% of the bone marrow’s cell population are characterised as MSCs, therefore, efforts to enrich the proportion of MSCs are under development. STRO-1 is a well-regarded cell surface antigen used in the characterisation of human MSC populations.^20^ Oreffo and colleagues have shown that by selecting with STRO-1, it is possible to enrich the MSC population during cell isolation.^21^ Further to this, Zannettino and coworkers^22^ have developed and characterised an analogous ovine marker, STRO-4, demonstrating efficient enrichment of oMSCs and for this reason implemented in the current study.

Despite advances, the active control of stem cell behaviour remains a challenge once implanted in the body. Biomechanical forces are important stimuli for influencing stem cell behaviour and are known to have a profound effect on bone repair.^23,24^ Evidence of this is presented in the early work of Lanyon and colleagues, where it was shown that mechanical loading above a critical threshold resulted in significant new bone formation in a rat model.^25^ This has been further validated in a number of small and large animal models to better understand the mechanisms of adaptation to mechanical loading in bone.^26^ Despite our understanding of how mechanics affect tissue remodelling and repair, clinical translation of mechanical stimuli has not been fully achieved in vivo at a cellular level. As such, implanted therapeutic cells lack the crucial mechanical stimuli required to direct repair in a physiological manner.^27^ This is largely attributed either to the limited translational potential of in vitro mechanical conditioning systems, concerns of direct mechanical loading causing further damage to the injured bone or scaffold stress shielding. Furthermore, recent data has shown that mechanical pre-conditioning of cell-seeded constructs prior to implantation may result in less integration and remodelling in the repair site.^28^ MICA addresses this challenge by non-invasively applying pico-newton forces directly to implanted MNP-labelled cells from outside the body using an external magnetic array, thereby activating mechanotransduction pathways. Furthermore, we demonstrate in this study for the first time that we can control stem cell behaviour remotely through mechanical forces in a pre-clinical animal model.

MNPs are versatile translational tools demonstrating value in several biomedical applications including targeted gene/drug delivery, magnetic hyperthermia and now in regenerative medicine. MNPs have received FDA approval for use as biocompatible MRI contrast agents enabling improved diagnostics and treatment of orthopaedic injuries.^29^ MNPs benefit from their capacity for remote magnetic manipulation and therefore offer a new source of cell control.^30^ Concerns of safety are at the forefront of any MNP-based research. We have extensively investigated stem cell health and behaviour following MNP labelling with a range of MNPs demonstrating no adverse outcomes nor secondary uptake at optimised doses.^31,32^ In our hands, the viability and function of MSCs labelled with Nanomag revealed no detectable long-term cytotoxicity either in vitro or in an in vivo subcutaneous mouse model.^19^ What remains unknown is the effect of MNP-labelling on cellular viability once implanted into the harsh microenvironment of the injured site. Importantly, the addition of the MNP label and the magnetic gradient did not elicit further cell death beyond that seen in all experimental groups (MNP-labelled and unlabelled cells). Limited survival rate at the repair site has been documented in other studies and is a well-accepted limitation of the of the cell therapy industry.^33,34^ Loss of cell viability can be attributed to a number of factors including a harsh inflammation environment, anoikis (lack of cell adhesion to the ECM) and limited oxygen and nutrients levels, all creating a hostile microenvironment leading to cell death.^33,34^ Our results, showing a reduction in cell number at 2 days’ post implantation in all groups, support these findings.^34^

Our results demonstrate initial enhanced bone repair in MICA treated defects in five out of the six sheep when compared to the internal MICA-control contralateral defect. Histological and immuno-histochemical analysis may suggest that MICA leads to bone of greater maturity and architecture. Defects from all groups were shown to repair via the endochondral ossification pathway as can be seen by the glycosaminoglycan molecules labelled by the toluidine blue stain.^35^ This closely mimics the developmental pathways of bone responsible for long bones and axial skeletal growth during embryogenesis. The developmental pathway involves the initiation of a hypertrophic cartilage template which subsequently undergoes mineralisation and remodelling to form functional bone.^36,37^ Evidence of remnant cartilaginous tissue and hypertrophic chondrocytes were observed in all groups with signs of enhanced matrix mineralisation present in MSC groups. This strongly suggests that the presence of exogenous MSCs work to promote soft tissue callus mineralisation towards mature mineralised bone as determined by micro-CT analysis.^38^ In our findings, we show initial evidence that remote dynamic loading of implanted cells may further enhance maturation as seen by the increase in ALP staining within the newly formed bone extracellular matrix, with evidence of osteoid seams lining new trabecular-like bone structures which are otherwise not present in MICA-control defects.

A strong periosteal reaction was observed within all defects from all groups. We hypothesize that MICA activation may further enhance this reaction, with increased bone mass observed at the proximal region of the defect resulting in bone outgrowth across the defect in all MICA treated defects, including donor 7, the non-responder. This region is rich in collagen, embedded with osteopontin and osteocalcin proteins and home to a variety of host cells including osteoblasts, osteoclasts, chondrocytes and endogenous MSCs, all contributing to repair and remodelling. In line with previous data generated in an ex vivo chick femur model,^13,39^ we hypothesise that one potential mode for action for MICA is through a paracrine effect initiating the secretion of cytokines and soluble factors from exogenous delivered MSCs to recruit and activate endogenous therapeutic cells.^30^ This assertion was supported by the increase in alkaline phosphatase staining, a marker of active remodelling, which may account for the new bone detected within that region.

A time point of 13 weeks was chosen for this study to enable us to investigate the early phases of repair in a bone injury defect ovine model. The challenge at this early phase is taking into account the biological variation present in multiple sheep donors. Due to the low levels of growth in the repair site overall, the inherent differences in donors was evident and influenced our ability to show statistical significance using overall mean bone volume data (supplementary figure 2). To study this in more detail and highlight the significance of the study, we have chosen to compare animals both individually and grouped using internal controls for MICA in the contralateral defects. In addition, cell efficacy and variability between the donors is known to result in variable animal responses. Finally, using the internal controls ensures that matched donor cells are used for both experimental and control groups.

The biggest challlenge faced in this study was translating the underlying MICA technology to the ovine animal model in a manner that is closly aligned to the in vitro bioreactor system which generated the proof of concept data from Henstock^13^ and Kanczler et al.^19^. The sole purpose of this bioreactor was to deliver a defined magnetic field at an oscillation frequency of 1 Hz to MNP-labelled cells in culture, using a permanent magnetic array where cells are exposed to a maximum field strength of 25 mT.^13,40^ Achieving a similar field strength in vivo was problematic due to the increase in distance between the external magnet and the site of injury correlating to an exponential decrease in field strength. By mimicking this scenario in vitro using the MICA bioreactor, we were able to design a system compatible with the ovine model to confidently infer a force directly to MNP-labelled MSCs and manipulate the TREK-1 ion channel for downstream osteogenesis. These results can be translated to human orthopaedic conditions in the future with advances in electromagnetic technologies where penetration depths of greater than 2.5 cm can be achieved. Furthermore, customised and tailor-made orthopaedic cuffs can be designed to house the electromagnetic system and targeted to injuries of all sizes and extremities.

We recognise that a limitation of this study was the lack of control over magnet oscillation where reliance was placed on animal activity to physically move the array. Despite efforts to schedule stimulation periods at moments of peak animal activity, this could not be standardised across sheep nor over the duration of the study. This had further implications on stimulation times where the decision to activate cells for a period of 3 h in vivo, as opposed to the standard 1 h implemented in vitro, was taken to account for animal rest periods ensuring that cells were stimulated for at least 1 h in total. Further work is underway to define optimal magnetic dosing in vivo and develop a suitable means of controlling oscillation using a bandage across a repair site.

As the prospect of stem-cell based therapies begin to enter the clinic, researchers and clinicians are encouraged to account for variability in stem cell function within a given patient population.^41^ The therapeutic potential of MSCs amongst patients has been shown to vary significantly in terms of growth kinetics and differentiation potential with consequences on in vivo bone healing.^42^ In line with studies by De Boer and coworkers,^42^ we not only demonstrate donor dependent tri-lineage potential, but also donor-dependent responses to biomechanical stimuli in vitro. A striking outcome from this study, was the profound effect of MICA activation on donors with low osteogenic potential in vitro and the clear correlation between levels of responses between in vitro and in vivo studies. Given that the pathogenesis of non-unions can, in many cases, be related to impaired osteogenesis, this data suggests that MICA-activation of autologous MSCs from non-union patients could have a stronger osteogenic response leading to improved clinical outcomes.^43^ This response is supported by data published by Charoenpanich et al.,^44^ where uniaxial cyclic tensile strain was shown to dramatically enhance osteogenesis of human MSCs from osteoporotic patients compared to healthy patients. Although more work is required to further investigate this theory, we present the potential to incorporate a mechanism for dynamic loading into orthopaedic stem cells therapies and improve outcomes.

MICA further benefits from having a completely aligned in vitro system which could potentially be used to develop a predictive assay to determine “good” and “poor” responders prior to treatment. Whilst the predictive element of this technology was not incorporated into the design of the current study, the strong correlation between the in vitro response of oMSCs to MICA activation and ultimate in vivo bone repair for the same sheep supports the use of this approach as a predictive assay. For example, the only donor which responded poorly to MICA activation in vitro was donor 7 which demonstrated an impaired response to MICA in vivo as well. Also, donors 6 and 10 demonstrated greatest improvement in mineralisation as a result of MICA activation in vitro and were similarly shown to perform best in the in vivo study. Although this data is preliminary, it does offer the possibility that MSCs from patients can be pre-screened and, based on these results, the clinician could then define how successful a MICA therapy would be for a patient. Further work is required to fully validate this potential application.

In our short term pre-clinical model, we present evidence to suggest that MICA technology can be used to augment and control cell based therapies in this case for a potential wide array of orthopaedic and other clinical applications. The MICA system can be used to apply remote cell loading in a variety of cell-only and cell-seeded scaffolds with varying degrees of stiffness. This innovative approach enables cells within soft injectable hydrogels to be loaded in situ following implantation, which has not previously been possible due to the soft nature of the gels rendering them incapable of withstanding mechanical loading. Furthermore, from a regulatory standpoint, where one-step surgical techniques are recommended, MICA can be adapted to match such scenarios.^45^ Finally, considering future clinical applications, our pre-clinical study supports the observation that inter-individual variation needs to be considered to better design human trials and predictive models.^46^

## Methods

Reagents were purchased from Sigma Aldrich, UK unless otherwise specified.

### Animal experiments

Methods were conducted in accordance to the UK Home Office Regulations and protocols approved by the University of Nottingham Animal Welfare and Ethical Review Body. For all surgeries, animals were placed in lateral recumbency to allow access to the sternum and medial aspect of both hind legs.

#### Sheep

Nineteen healthy, English Mule ewes aged 2–4 years with a mean weight of 77 kg were used and assigned randomly to each treatment groups (Table 1). It should be noted that each sheep received a different treatment in each leg.

#### Bone marrow harvest

Autologous MSCs were isolated by bone marrow aspiration from the sternum of anesthetized animals using a 100 mm 8 Gauge Jamshidi needle, (UK Medical Ltd., Sheffield, UK). Aspirate was collected in αMEM containing 10% FBS, 1% L-glutamine, 1% antibiotic and anti-mycotic (AA) and heparin sodium to prevent clotting (5000 IU/ml, Wockhardt, Wrexham, UK).

#### Defect

Three weeks post initial bone marrow harvest, a single cylindrical defect (8 mm diameter × 15 mm deep) was created in the cancellous bone region of the medial femoral condyle in the left and right hind leg of each animal. Throughout coring and reaming, the drills were cooled with sterile saline solution to prevent tissue damage.

#### Cell delivery

Pre-set ECM constructs were immediately implanted within the defect using the customised delivery device (supplementary data, Fig. 1).

#### Sheep truss

Twenty four hour post defect surgery, sheep were fitted with the modified truss and either the magnetic array or the sham array aligned to the location of the defect. Trusses were worn for 3 h/day, 5 days/week.

#### Sacrifice

Sheep were sacrificed either at 2 days (experiment 1) or 13 weeks (experiment 2) post-op by pentobarbital overdose administered intravenously. The femoral condyles were retrieved immediately and trimmed for further analysis (Micro-CT and histology). Samples were fixed in 10% neutral buffered formalin for 7 days before proceeding.

### Selection of STRO-4 positive MSCs

The mononuclear cell fraction from each donor was isolated by red blood cell (RBC) lysis treatment by initially filtering the aspirate using a 100 µm cell sieve and centrifuging (220 g; 30 min). The supernatant was carefully removed, replaced with 2 ml of ice cold RBC lysis buffer and incubated (3 min; RT) with gentle agitation. Lysis buffer was quenched with 45 ml ice cold PBS and lysed cells removed by centrifugation (220 g; 5 min). This process was repeated until a white pellet appeared at which point 2 ml of blocking buffer (αMEM, 10% rat serum, 1% bovine serum albumin (BSA) and 5% FBS) was added to the pellet and incubated (30 min; 4 °C). Cells were then washed with MACS buffer (PBS, 0.5% BSA and 2 mM EDTA disodium salt) and incubated with the STRO-4 IgG hybridoma (20 µg/ml; *Adelaide University)* for 30 min at 4 °C. Cells were again washed with MACS buffer and incubated with 200 µl of the MACS anti-mouse IgG MicroBeads *(Miltenyi Biotec, UK)* (30 min; 4 °C) prior to MACS separation using the LS MACS column *(Miltenyi Biotec, UK)*. STRO-4 oMSCs were collected and plated in expansion media (αMEM media, 20% FBS, 1% L-Glutamine and 1% AA) and maintained at 37 °C for 1 week before further media changes. STRO-4 positive oMSCs were cultured under standard cell culturing conditions in αMEM (10% FBS, 1% L-glutamine and 1% AA).

### MNP labelling of STRO-4 positive oMSCs

Nanomag *(Micromod, Germany)*, a commercially available 250 nm, carboxyl-coated MNP was functionalised with a TREK-1 antibody *(Alomone Labs, APC-047, Israel)* as described previously.^13^ To label oMSCs, cells at 80–90% confluency were trypsinized, counted and washed in PBS to remove any residual FBS. Cells were then re-suspended in serum free media (SFM) and incubated with TREK-1 functionalised MNPs (1 mg/ml) at a ratio of 25 µg MNPs per 10^6^ cells with 1 µl DOTAP (1 µg/ml) (3 h; 37 °C). The corresponding unlabelled cell groups were simultaneously incubated in SFM only. Unbound MNPs were removed and cells washed in PBS by centrifugation (1000 rpm; 5 min).

### Encapsulation of oMSCs within a ECM gel construct for in vivo delivery

Preparation of the ECM digest (12.5 mg/ml) is described in a previously published article.^47^ In brief, 5 × 10^6^ MNP labelled or unlabelled oMSCs from each donor were re-suspended in a 20% HEPES solution (prepared in SFM) and thoroughly mixed with the ECM digest at a ratio of 1:3 to achieve a final volume of 0.8 ml. The subsequent gel mixture was then transferred to a customised sterile delivery device and allowed to set for 1 h at 37 °C before hydrating with 500 µl SFM. Pre-set constructs were maintained at 37 °C and implanted the following day. Acellular constructs were prepared in a similar manner.

### In vitro donor response to MICA activation

Donor response to MICA activation was assessed in vitro using a collagen hydrogel system previously reported.^13^ Here, 2.5 × 10^5^ oMSCs (P3) from each donor were encapsulated in 3.94 mg/ml stock solution of rat tail type 1 collagen and neutralised with a 20% HEPES solution (prepared in SFM) to a final concentration of 2.5 mg/ml and volume of 300 µl. The collagen and cell suspension was seeded into non-adherent 48-well plates and allowed to set (1 h; 37 °C) before hydrating with 1 ml of osteogenic differentiation media. Hydrogels were cultured for 28 days in osteogenic media with a single media change per week. MICA groups consisted of MNP-labelled cells and were stimulated for 1 h/day in the MICA bioreactor while the static groups consisted of unlabelled cells and were maintained in identical conditions without a magnetic field. Mineralisation levels were evaluated by micro-CT (micro-CT 50, Scanco, Switzerland) on day 28. Micro-CT scans were performed with beam energy of 55 kVp, intensity of 145 µA, a 200 ms integration and spatial resolution of 10 µm.

### Assessment of cellular viability by LDH staining

#### Construct preparation

ECM constructs were prepared as described above and implanted immediately within the femoral defect. Donor matched in vitro controls consisting of unlabelled oMSCs were simultaneously prepared and maintained in culture for the duration of the study (2 days). SFM in control groups was changed to expansion media at the time of in vivo implantation.

#### Construct harvest

Implanted ECM constructs were harvested from the defect of sacrificed sheep, transferred directly to expansion media to maintain cell viability and transported on ice. Constructs (implanted and in vitro controls) were embedded in optimum cutting temperature (OCT) medium (VWR, UK) and frozen by immersing in liquid nitrogen cooled isopentane and stored at −20 °C until cryosectioning (Bright, Clinicut Clinical Cryostat).

#### LDH staining

Viable cells were identified post implantation by the presence of the active LDH enzyme. Sections (16 µm) were incubated in a staining solution consisting of 7.2 mg/dL NBT (nitro blue tetrazolium; Fisher Scientific) and 60 mg/dL NADH (β-Nicotinamide adenine dinucleotide hydrate) prepared in 0.05 M TRIS buffer at pH 7.6. (30 min; 37 °C). Unused reagents were removed by a single water wash and then in ascending and descending concentrations of acetone (30, 60, 90%). Slides were mounted with Hydromout and imaged (Nikon Eclipse, Ti-S). Implanted cells were identified by red fluorescence (CM-DiI staining) and viable cells by blue staining under bright field settings. Ten random field of views were imaged per section in a total of five sections. Viability was evaluated by ImageJ by quantifying the proportion of dual LDH and CM-DiI staining relative to total CM-DiI staining.

### Micro-computed tomography (Micro-CT) evaluation of bone repair at 13 weeks

Bone growth was determined by micro-CT (Skyscan 1174, Skyscan, Kontich, Belgium). Micro-CT scans were performed with beam energy of 50 kV, current of 800 µA, 0.50 µm aluminium filter and a voxel resolution of 32 µm. A threshold of 255/50 was selected to segment bone from surrounding tissue and includes both mineralised bone and immature bone. Transmission images were reconstructed using Skyscan supplied software (NRecon) with the resulting 2D image representing a single 32 µm slice (1/256).

### Statistical analysis

GraphPad Prism 6.0 was used for all statistical assessments. In most cases, data is presented as the average value ± standard deviation (S.D.) unless otherwise stated. Figure 1b: In vitro donor response to MICA activation. Significance was determined by two-way ANOVA with a post hoc Sidaks multiple comparison test (Alpha = 0.05). Figure 2a: Determining the minimum magnetic field strength required for cell activation. Data here, represents the mean value ± SEM with significance determined by one-way ANOVA and a Dunnetts multiple comparison test (Alpha = 0.05). Figure 2c: Quantification of bony nodules. Significance was determined by one-way ANOVA with a post hoc Tukey test (Alpha = 0.05). Figure 3c**:** Quantification of cellular viability. Significance was determined by one-way ANOVA test with a post hoc Tukey test (Alpha = 0.05). Figure 4b, c: Averaged percentage change in bone fill. Data represents the average percentage change in bone fill ± SEM with significance determined by a one-way ANOVA with a post hoc Tukey test (Alpha 0.05). Figure 5a, b: Total bone formation. Data represents the average total bone volume ± SEM with significance determined by a two-way paired *t* test. In all cases; *P < 0.05, **P < 0.01, ***P < 0.001, ****P < 0.0001 and ns is no significance and data is considered to be normally distributed except micro-CT data.

For further method detail please refer to the “Supplementary Methods” section in supplementary information.

### Data availability

The data sets generated during and/or analysed during the current study are available from the corresponding author on reasonable request.

## Electronic supplementary material

Supplementary Material

Supplementary Figure 1

Supplementary Figure 2

Supplementary Figure 3

## References

[1] Stevens MM (2008). Biomaterials for bone tissue engineering. Mater. Today.

[2] Meng J (2013). Super-paramagnetic responsive nanofibrous scaffolds under static magnetic field enhance osteogenesis for bone repair in vivo. Sci. Rep..

[3] Cancedda R (2007). A tissue engineering approach to bone repair in large animal models and in clinical practice. Biomaterials.

[4] Gaston M (2007). A. H. R. W. Inhibition of fracture healing. J. Bone Jt. Surg. Br. Vol..

[5] Gothard D (2014). Tissue engineered bone using select growth factors: a comprehensive review of animal studies and clinical translation studies in man. Eur. Cell. Mater..

[6] Dimitriou R (2011). Bone regeneration: current concepts and future directions. BMC Med..

[7] Pape H (2010). Bone defects and nonunions–What role does vascularity play in filling the gap?. Injury.

[8] Salgado A (2004). Bone tissue engineering: state of the art and future trends. Macromol. Biosci..

[9] Markides H (2015). Overcoming translational challenges - The delivery of mechanical stimuli in vivo. Int. J. Biochem. Cell Biol..

[10] Black CR (2015). Bone tissue engineering. Curr. Mol. Biol. Rep..

[11] Raisz LG (1999). Physiology and pathophysiology of bone remodeling. Clin. Chem..

[12] Liedert A (2006). Signal transduction pathways involved in mechanotransduction in bone cells. Biochem. Biophys. Res. Commun..

[13] Henstock JR (2014). Remotely activated mechanotransduction via magnetic nanoparticles promotes mineralization synergistically with bone morphogenetic protein 2: applications for injectable cell therapy. Stem Cells Transl. Med..

[14] Liedert, A. et al. Mechanobiology of Bone Tissue and Bone Cells. *In**Mechanosensitivity in Cells and Tissues*. (eds Kamkin A., Kiseleva I.) https://www.ncbi.nlm.nih.gov/books/NBK7494/ (Academia, Moscow, 2005). 21290762

[15] Cartmell SH (2002). Mechanical conditioning of bone cells in vitro using magnetic micro particle technology. Eur. Cells Mater..

[16] Hughes S (2007). Magnetic targeting of mechanosensors in bone cells for tissue engineering applications. J. Biomech..

[17] Hughes S (2008). Selective activation of mechanosensitive ion channels using magnetic particles. J. R. Soc. Interface.

[18] Hughes S (2005). Magnetic micro- and nanoparticle mediated activation of mechanosensitive ion channels. Med. Eng. Phys..

[19] Kanczler J (2010). Controlled differentiation of human bone marrow stromal cells using magnetic nanoparticle technology. Tissue Eng. Part. A..

[20] Lv F (2014). Concise review: the surface markers and identity of human mesenchymal stem cells. Stem Cells.

[21] Williams EL (2013). Isolation and enrichment of Stro-1 immunoselected mesenchymal stem cells from adult human bone marrow. Methods Mol. Biol..

[22] Gronthos S (2009). Heat shock protein-90 beta is expressed at the surface of multipotential mesenchymal precursor cells: generation of a novel monoclonal antibody, STRO-4, with specificity for mesenchymal precursor cells from human and ovine tissues. Stem. Cells Dev..

[23] Weaver AS (2010). The effects of axial displacement on fracture callus morphology and MSC homing depend on the timing of application. Bone.

[24] Scott A (2008). Mechanotransduction in human bone: in vitro cellular physiology that underpins bone changes with exercise. Sports Med..

[25] Rubin CT (1985). Regulation of bone mass by mechanical strain magnitude. Calcif. Tissue Int..

[26] Meakin LB (2014). The contribution of experimental in vivo models to understanding the mechanisms of adaptation to mechanical loading in bone. Front Endocrinol. (Lausanne).

[27] Pioletti DP (2010). Biomechanics in bone tissue engineering. Comput. Methods Biomech. Biomed. Engin..

[28] Lyons FG (2010). The healing of bony defects by cell-free collagen-based scaffolds compared to stem cell-seeded tissue engineered constructs. Biomaterials.

[29] Markides H (2013). Whole body tracking of superparamagnetic iron oxide nanoparticle-labelled cells–a rheumatoid arthritis mouse model. Stem Cell Res. Ther..

[30] Wilhelm C (2008). Universal cell labelling with anionic magnetic nanoparticles. Biomaterials.

[31] Harrison R (2016). Autonomous magnetic labelling of functional mesenchymal stem cells for improved traceability and spatial control in cell therapy applications. J. Tissue Eng. Regen. Med..

[32] Mahmoudi M (2011). Toxicity evaluations of superparamagnetic iron oxide nanoparticles: cell “vision” versus physicochemical properties of nanoparticles. ACS Nano.

[33] Lee S (2015). Cell adhesion and long-term survival of transplanted mesenchymal stem cells: a prerequisite for cell therapy. Oxid. Med. Cell Longev..

[34] Deschepper M (2011). Survival and function of mesenchymal stem cells (MSCs) depend on glucose to overcome exposure to long-term, severe and continuous hypoxia. J. Cell.

[35] Bornes TD (2014). Mesenchymal stem cells in the treatment of traumatic articular cartilage defects: a comprehensive review. Arthritis Res. Ther..

[36] Bardsley K (2017). Repair of bone defects in vivo using tissue engineered hypertrophic cartilage grafts produced from nasal chondrocytes. Biomaterials.

[37] Knight MN (2013). Mesenchymal stem cells in bone regeneration. Adv. Wound Care.

[38] Thompson EM (2016). An endochondral ossification-based approach to bone repair: chondrogenically primed mesenchymal stem cell-laden scaffolds support greater repair of critical-sized cranial defects than osteogenically stimulated constructs in vivo. Tissue Eng. Part. A..

[39] Saeed H (2016). Mesenchymal stem cells (MSCs) as skeletal therapeutics–an update. J. Biomed. Sci..

[40] Hu B (2013). Receptor-targeted, magneto-mechanical stimulation of osteogenic differentiation of human bone marrow-derived mesenchymal stem cells. Int. J. Mol. Sci..

[41] Kretlow JD (2008). Donor age and cell passage affects differentiation potential of murine bone marrow-derived stem cells. BMC Cell Biol..

[42] Siddappa R (2007). Donor variation and loss of multipotency during in vitro expansion of human mesenchymal stem cells for bone tissue engineering. J. Orthop. Res..

[43] Csongradi JJ (1989). Ununited lower limb fractures. West. J. Med..

[44] Charoenpanich A (2014). Cyclic tensile strain enhances osteogenesis and angiogenesis in mesenchymal stem cells from osteoporotic donors. Tissue Eng. Part. A..

[45] Ma J (2014). Concise review: cell-based strategies in bone tissue engineering and regenerative medicine. Stem Cells Transl. Med..

[46] Glueck M (2015). Induction of osteogenic differentiation in human mesenchymal stem cells by crosstalk with osteoblasts. Biores. Open Access.

[47] Sawkins MJ (2013). Hydrogels derived from demineralized and decellularized bone extracellular matrix. Acta Biomater..

