# Supplementary material for: Translation of remote control regenerative technologies for bone repair
Source: NPJ Regen Med. 2018 Apr 17;3:9. doi: 10.1038/s41536-018-0048-1 (PMC5904134; doi:10.1038/s41536-018-0048-1)
Supplement: Supplementary file 1 — Supplementary Material [file 41536_2018_48_MOESM1_ESM.docx]

**Supplementary Information**

**Supplementary Methods.**

1. **Animals**

Methods were conducted in accordance to the UK Home Office Regulations and protocols approved by the University of Nottingham Animal Welfare and Ethical Review Body. Nineteen female non-pregnant skeletally mature (age 2 - 4 years, weight 64.5 - 89.5 kg) English Mule sheep were used. A power calculation (GenStat (Release 15.2)) was carried out to select the absolute minimum number of animals needed to allow for biological variability. Sheep were randomly allocated to each experimental group to minimise bias and were assigned to groups by independent animal technicians. Animals were screened to ensure good physical condition and were acclimatised to the new environment for a minimum of 7 days prior to surgery. After surgery, animals were housed in individual pens for 24 hrs and then housed in a group pen. Prior to surgery food was withheld for a minimum of 12 hrs, but water was available ad libitum.

- 1. **Anaesthesia and sustained release analgesia**

Sheep were pre-medicated intramuscularly with 2% Rompun (0.1 mg/kg Xylazine, Bayer, Newbury, UK). Anaesthesia was induced with Ketaset (Ketamine, 2 mg/kg, Fort Dodge Animal Health Ltd, Southampton, UK) and 2.5 mg Hypnovel (Midazolam, Roche Products Ltd, Welwyn Garden City, UK), and then maintained with 2% Isoflurane (Abbott Laboratories Ltd, Maidenhead, UK) in 100% oxygen. Pre- and post-operative analgesia was given via sustained release Durogesic 75 mcg/hr patches (Fentanyl, Janssen-Cilag, Saunderton, UK).  Prophylactic antibiotics (Duphapen and Streptomycin, Pfizer, Kent, UK, 1 ml/25 kg intramuscularly) were given pre-operatively and 24 hrs post-operatively.

**1.2 Sternal bone marrow aspiration.**

Sheep were placed in lateral recumbency to allow access to the sternum.  The sternum was then clipped and prepared using a chlorhexidine gluconate solution BP 20% (Hibiscrub Veterinary, Schering-Plough, UK) followed by an alcohol gel before surgical drapes were applied.  A 100 mm 8 Gauge Jamshidi needle (UK Medical Ltd., Sheffield, UK) was inserted into the bone marrow of the 3rd-5th sternebrae.  The guide was then removed and a 50 ml luer lock syringe, coated with a 0.5% solution of heparin sodium (5000 IU/ml, Wockhardt, Wrexham, UK).  A pumping action was applied to remove a maximum of 15 ml of aspirate from 3 separate locations on the sternum. After completion, a local anaesthetic (Lidocaine hydrochloride, Hameln Pharmaceuticals, Gloucester, UK) was delivered to the sites and a moisture vapour - permeable spray dressing (Opsite, Smith & Nephew Healthcare, Hull, UK) was then applied over the wound to protect it.

**1.3 Bone Defect Surgery**

Animals were placed in lateral recumbency to allow access to the medial aspect of both hind legs. The area was clipped and prepared using chlorhexidine gluconate solution BP 20% (Hibiscrub Veterinary, Schering-Plough, UK). Lidocaine (Hameln Pharmaceuticals, Gloucester, UK) was given as a local anaesthetic around the incision site. Once in theatre, the area was thoroughly washed with Virusan Gel containing Triclosan 0.05% w/v (Amity International Healthcare, Barnsley, UK). A stomach tube was placed for the duration of the surgery and Hartmann’s solution (Vetivex™11, Dechra, Shrewsbury, UK) was given intravenously.

A drill hole was made in the cancellous bone of the medial femoral condyle in both the left and right hind leg. An 8 mm wide bone corer in combination with a guide was used to make an initial cut in the bone and was followed by an 8 mm diameter x 15 mm deep cylindrical hole created using an 8mm wide bone corer with a 15 mm stop. The bone core was then removed and an 8mm wide reamer with a 15 mm stop was used to ensure that the defect was standardised. Throughout coring and reaming the drills were cooled with sterile saline solution. The defect was then thoroughly washed with sterile saline solution and then dried with a sterile swab before the cell seeded construct was delivered to the defect. The subcutaneous tissue and skin were closed using resorbable sutures (Vicryl 0, Ethicon, Kirkton, UK). Lidocaine was then reapplied to the defect site to provide additional local analgesia and a moisture vapour - permeable spray dressing (Opsite, Smith & Nephew Healthcare, Hull, UK) applied over the wound to protect it.

**1.4 Accelerometers**

To assess the activity patterns of the sheep a subsection of the study group wore a unaxial accelerometer attached to a collar on the sheep perpendicular to the plane of forward movement and a record logged every 30 sec over a 24 hr period. Data were downloaded and processed using Actiwatch Activity and Sleep Analysis software (Version 5.25, Cambridge Neurotechnology Limited, Cambridge, UK).

1. **CM-DiI labelling**

Cells were labelled with a fluorescent cell tracker dye, CM-DiI prior SPION labelling and ECM encapsulation (experiment 1, table 1).  A stock solution of CM-DiI (Molecular Probes, Paisley UK) was prepared in dimethyl sulfoxide (DMSO) at a concentration of 1 mg/ml. oMSCs in suspension were incubated in the CM-DiI working solution (3 mM) for 5 min at 37°C, and then for an additional 15 min at 4°C, away from light. Unincorporated dye was removed via centrifugation (300 g; 5 min) and washed with PBS before re-suspending in SFM for SPION labelling and delivery.

1. **Magnet array fabrication and validation**

Magnetic arrays were manufactured at the North Staffordshire Hospital Medical Workshop and consisted of an aluminium frame with machined inserts to securely position each magnet (Neodymium iron boron magnetic purchased form www.e-magnetsuk.com) and 4 mild steel back plates to shield from stray magnetic fields. The final array used consisted of 2 (4x2x1 cm) magnets (EP352) placed at opposing poles to each other. The average magnetic field strength of each array was measured using a Guassmeter positioned in 6 locations across the surface of each magnet at 3 distances (0, 1.2 and 2.5 cm). Magnets were replaced with no-magnetic aluminium blocks of equal dimensions and weight to create sham arrays.

1. **HEK 293 cells**

Commercially sourced HEK-293 NFK-B luciferase reporter cell line was used to determine the minimum magnetic field required to mechanically activate cells. Here, cells were seeded in monolayer at a cell seeding density of 2x10^4^ cells/cm^2^ and allowed to attach overnight. Cells were then serum starved for 1hr prior labelling with TREK-functionalised MNPs (25µg/2x10^4^ cells) and DOTAP for 1.5 hrs. Labelled cells were then stimulated for 1hr using the MICA bioreactor at increasing distances from the magnet (3.8, 2.8, 2.5, 1.8. 0.7 cm). Media was collected 3hr post stimulation and secreted luciferase determined by Pierce™ Gaussia Luciferase Flash Assay Kit (ThermoFisher Scientific, UK) as recommended by the supplier.

1. **Tri-lineage differentiation of ovine mesenchymal stem cells (oMSCs)**.

STRO-4 positive oMSCs (P3) were characterised by their ability to undergo differentiation towards the osteogenic, adipogenic and chondrogenic lineages. ***Osteogenic differentiation***; Cells were plated in triplicate at a cell seeding density of 10^4^ cells/cm^2^ and treated with osteogenic induction media consisting of low glucose DMEM (1 g/L), 10% FBS, 1% L-glutamine, 1% AA, 10^-8^mM dexamethasone, 0.8 mM L-Ascorbic Acid and 10mM β-glycerophosphate*.* Cells were cultured for 21 days and fixed in 10% neutral buffered formalin (10 min; RT) for Alizarin red staining (1%). ***Adipogenesis;*** Cells were seeded at a density of 2.5x10^4^ cells/cm^2^ in triplicate and cultured in induction media consisting of high glucose DMEM (4.5 g/L), 1% BSA, 100 µM indomethacin, 1 µm dexamethasone, 0.5 mM IBMX (3-Isobutyl-1-methylxanthine) and 10 µg/ml insulin for 72 hrs. Cells, thereafter were cultured in adipogenic maintenance media for a further 14 days. Adipogenesis was evaluated by oil red O staining. ***Chondrogenesis*** was induced in 3D pellet cultures. In brief, 4x10^5^ cells were pelleted in 1.5 ml Eppendorf tubes and centrifuged at 0.3 g for 5 min in chondrogenic media containing, high glucose DMEM (4.5 g/L), 1% FBS, 1% L-glutamine, 1% AA, 0.1 µm dexamethasone, 50 µg/ml L-Ascorbic Acid, 10 ng/ml TGF-β1 (Peprotech, UK) and 50 mg/ml ITS (insulin, transferrin, sodium selenite). Media was completely changed every 3 days for 21 days. Chondrogenesis was evaluated by histologically by Alcian blue staining. In all cases, control cells were cultured in proliferation media for the duration of the protocol.

1. **Histology**

***Decalcified histology;*** Bones were decalcified by formic acid treatment at 4 °C prior to paraffin embedding. Coronal sections (10 µm thickness) from the central area of the defect were cut and stained for Masson-Goldner trichrome (Merck-Millipore, UK), Pricrosirus red, Toluidine blue and Alcian blue. Sections were then mounted in DPX and imaged (EVOS). ***Calcified histology;*** Bones were placed into 50% ethanol then 75% ethanol for 1 week each and then placed in LR White Hard Resin (London Resin Company Ltd, Berkshire, UK) for 3 weeks under vacuum, with fresh resin changes weekly. Blocks were polymerised using LR White accelerator at -10°C and then stored at room temperature until processed.

***Immunohistochemistry.***

Coronal (10 µm thick) sections were deparaffinised and rehydrated prior to antigen retrieval (0.1% trypsin made up in 1% calcium chloride; 10 min incubation at 37 ^o^C). Endogenous peroxidases were quenched (3% hydrogen peroxide made up in 50% methanol; 5 min incubation at RT) and sections subsequently blocked with 3% BSA for 1hr at RT). Primary antibodies, ALP (0.5 µg/ml, Santa Cruz, Sc365765), Osteocalcin (10 µg/ml, Abcam, ab13420) and Osteopontin (10 µg/ml, Biobryt, Orb222555) were added and incubated at 4^o^C, overnight. Upon thorough washing, sections were stained using the Biotinylated ABC kit (Abcam, UK) and DAB substrate (Abcam, UK) as specified by the manufactured. Finally, sections were dehydrated and mounted in DPX).

***
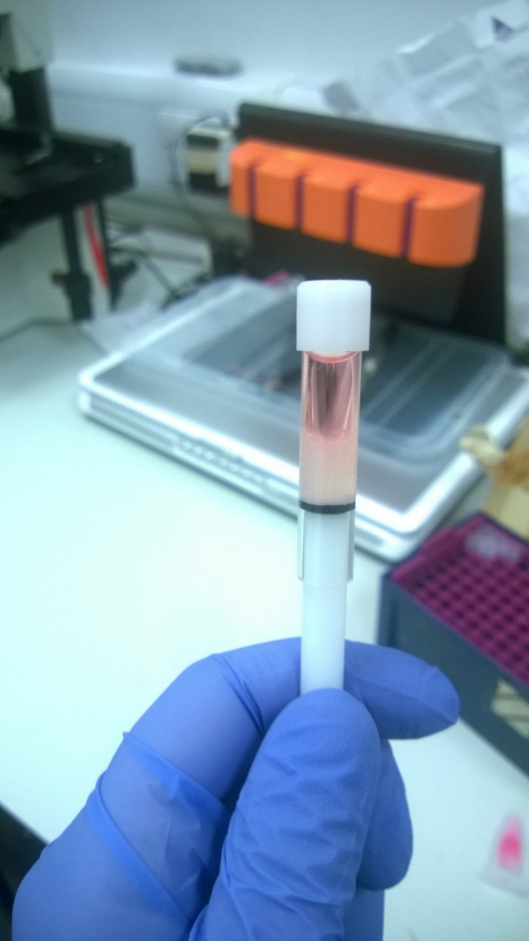
***

**Supplementary figure 1:** Device to deliver pre-set cell seeded ECM construct

**Supplementary Figure 2**

The gold standard treatment for large skeletal defects is typically autologous bone graft and therefore represents the positive control in this study. This treatment group resulted in significantly enhanced levels of bone formation compared to all other groups (p<0.01). Considering the average population response, all cell-based therapy groups demonstrated a slight but insignificant increase in bone growth over the empty defect with MICA resulting in 7-12% more bone growth than the MICA-control groups. Similar levels of bone growth were observed for the ECM carrier group.

**
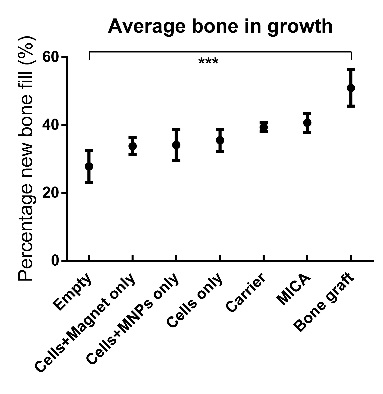
**

**Supplementary Figure 2:** Micro-CT evaluation of bone repair at 13 weeks’ post implantation. Graph representing the average bone fill (%) for all groups. Data represents the average bone volume for each group ± SEM with significance determined by a non-parametric Kruskal-Wallis with a post-hoc Dunns multiple comparisons test, due to non-normally distributed data.

**Supplementary Figure 3**


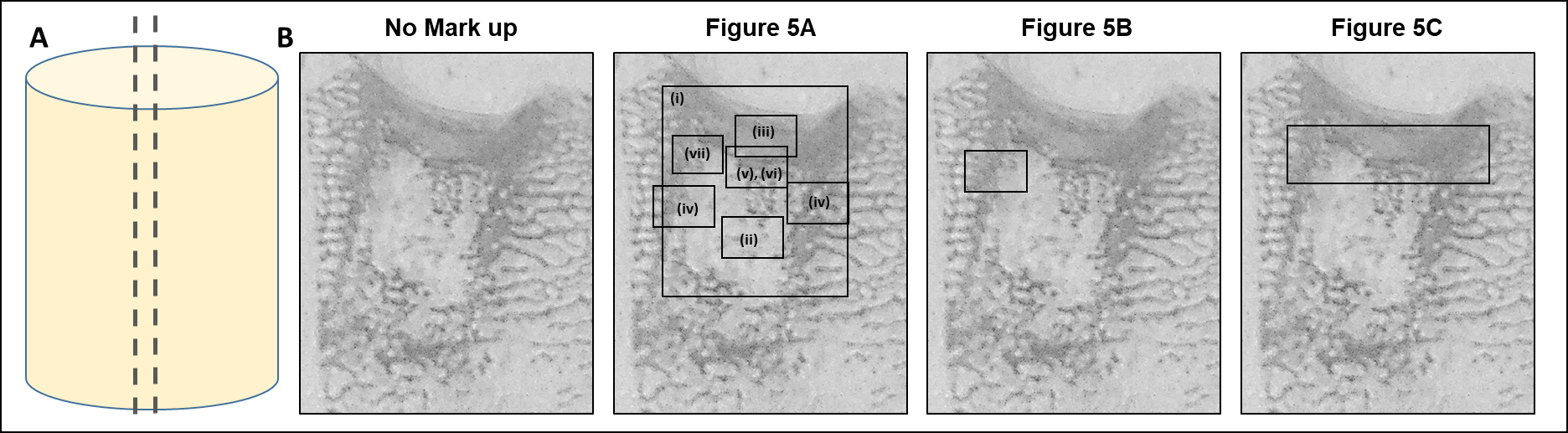


**Supplementary Figure 3:** (A) Schematic highlighting the region through the defect where all sections were taken B) representative image of a defect to demonstrated anatomical location of each section in Figure 6.
