# Supplementary figures and images for: Translation of remote control regenerative technologies for bone repair
Source: NPJ Regen Med. 2018 Apr 17;3:9. doi: 10.1038/s41536-018-0048-1 (PMC5904134; doi:10.1038/s41536-018-0048-1)

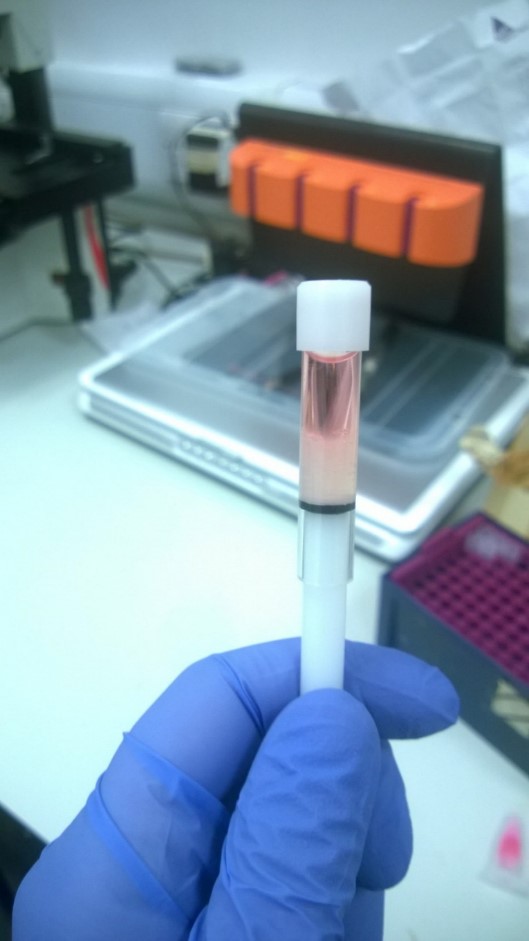

Supplement: Supplementary file 2 — Supplementary Figure 1 [file 41536_2018_48_MOESM2_ESM.jpg]

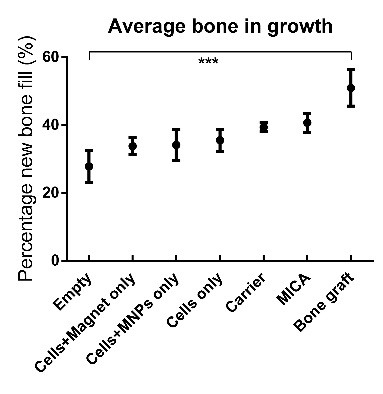

Supplement: Supplementary file 3 — Supplementary Figure 2 [file 41536_2018_48_MOESM3_ESM.jpg]

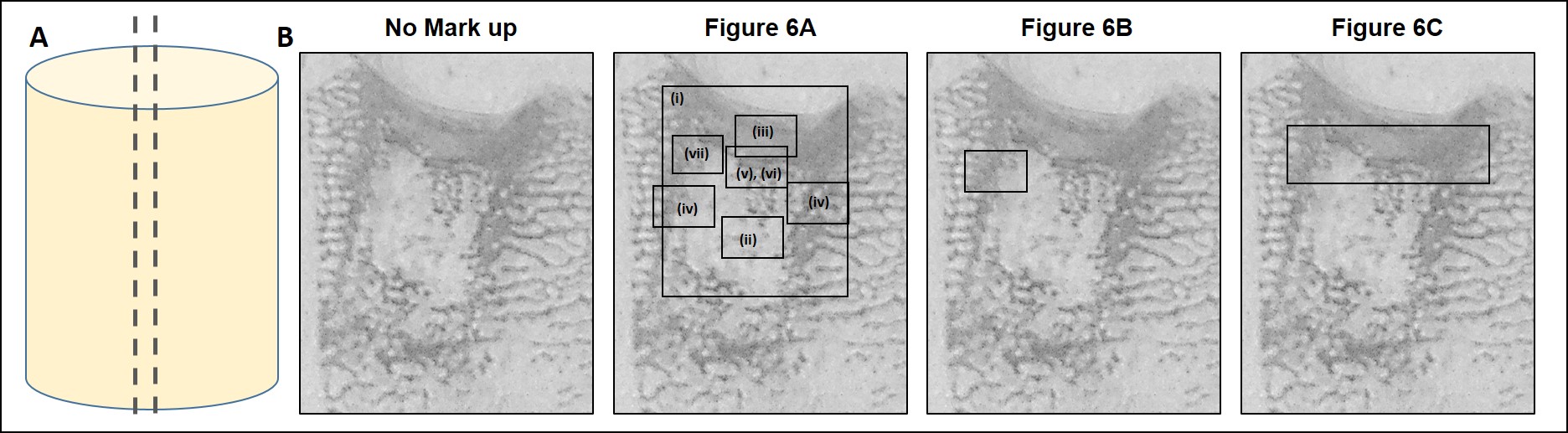

Supplement: Supplementary file 4 — Supplementary Figure 3 [file 41536_2018_48_MOESM4_ESM.jpg]
